# Professor Felix Plateau

**Published:** 1911-04-01

**Authors:** 


Professor Felix Plateau, Emeritus Professor of the University of Ghent, recently died at the age of seventy. In 1871 he was appointed an extraordinary professor, and in 1875 was promoted full professor in the Faculty of Science, where he lectured on zoology and comparative anatomy. His lectures were always clear and methodical, and in private life he was of a modest and retiring disposition. He was a member of the Academy of Sciences of Belgium and a Commander of the Order of Leopold.

